# Community Perspectives on Zika Virus Disease Prevention in Guatemala: A Qualitative Study

**DOI:** 10.4269/ajtmh.19-0578

**Published:** 2020-02-24

**Authors:** Elli Leontsini, Sean Maloney, Margarita Ramírez, Luisa María Mazariegos, Elisa Juárez Chávez, Diana Kumar, Priya Parikh, Gabrielle C. Hunter

**Affiliations:** 1 Department of International Health, Johns Hopkins Bloomberg School of Public Health, Baltimore, Maryland;; 2 Johns Hopkins Center for Communication Programs, Baltimore, Maryland;; 3 Independent Consultant, Guatemala City, Guatemala

## Abstract

Zika virus, which is transmitted by *Aedes aegypti* mosquitoes and through sexual transmission, disproportionally affects the human fetus. Guatemala experienced a surge of Zika cases beginning in 2016. We conducted a qualitative study of community perceptions of the seriousness of Zika, as well as the effectiveness, feasibility, and collective efficacy of Zika prevention actions. Free listing elicited the preventive actions salient for 68 participants comprising pregnant women, men with a pregnant partner, and women likely to become pregnant; 12 focus group discussions in a highland and a lowland town explored other concepts through rank orderings of prevention practices depicted on cards. Participants’ initial concern about Zika, based on recent experience with chikungunya and high media coverage, diminished because of its mild symptoms and reduced media coverage. Participants identified more than 32 salient preventive actions, many of which are considered effective by programs. Participants ranked water storage container cleaning and regular unspecified cleaning of the house and its surroundings as highly effective, feasible, and of high collective efficacy; however, the actions lacked the specificity needed to effectively destroy mosquito eggs. Community-level removal of tires and discarded containers had lower collective efficacy than household-level implementation because of the municipal and community cooperation needed. Condom use, although salient for Zika prevention, was hindered by gender roles. The findings indicate space for increasing self-efficacy for condom use among fathers-to-be, abandoning nonspecific terms such as “cleaning” and “standing water,” increasing people’s skills in using bleach as an ovicide, and promoting antenatal care and family planning counseling.

## INTRODUCTION

Zika is the latest known arbovirus transmitted by the *Aedes aegypti* mosquito in the Americas. The first cases of the disease were identified starting in May 2015 in Brazil, and they expanded rapidly through the vector’s range in the region because of the lack of population immunity.^1^ This variety of *Ae. aegypti* can also transmit yellow fever, dengue, and chikungunya, which appeared historically in that order, in the Americas.^2^ *Aedes aegypti* is diurnal and bites its human targets indoors or in shaded outdoor areas around the home.^3^ Its eggs are laid just above the waterline in human-generated containers, where they remain dormant until the water level rises, and they stay protected when water pools dry up.^2^ In Guatemala, households typically store water in large drums and cement laundry washbasins, referred to as *pilas*. Zika typically has mild or no symptoms, but it disproportionately affects the fetuses of pregnant women, causing neurological birth defects.^4^ There is no vaccine or treatment for Zika. In addition to the mosquito-borne route of infection, Zika is also sexually transmitted.^5^ The WHO, therefore, pronounced Zika a public health emergency of international concern from February to November 2016 and an endemic health problem in Latin America and the Caribbean (LAC) ever since.^6^

Guatemala experienced a surge of Zika cases in 2015 and 2016,^7^ and cases continued to be reported afterward, with 1,032 confirmed cumulative cases and 140 confirmed cases of congenital syndrome associated with Zika until 2017.^8^ Since that time, national health authorities and local organizations began to provide information about the threat of Zika and to recommend preventive actions to take at the individual, household, and community levels. These actions can be categorized as follows: actions to avoid mosquito bites, actions to eliminate mosquitoes at various life stages (eggs, larvae, pupae, and adults in and around the home), and actions to prevent the sexual transmission of Zika. Emphasis is placed on protecting women during pregnancy. In addition, enabling behaviors such as seeking prenatal care and family planning counseling are promoted.^9^

The Center for Communication Programs funded by the U.S. Agency for International Development (USAID) provided technical assistance in the LAC region since 2016, including in Guatemala, advising the development of national Zika communication strategies.^10^ Ample information has been collected on Zika knowledge and risk perception,^11–16^ but information on the community’s sense of the efficacy of prevention behaviors is scarce.^17–19^ In this qualitative study, we aimed to obtain much needed contextual data for further improving social and behavior change communication (SBCC) efforts. We aimed to gain insights into five behavioral determinants among study participants in Guatemala: the perceived seriousness of Zika illness and how it may have changed over the course of the outbreak, the cultural salience of freely elicited actions that people in the community take to avoid Zika, the relative effectiveness and feasibility of Zika prevention recommendations, and relative collective efficacy to practice them in the community context.

## METHODS

### Guiding concepts.

The extended parallel processing model^20^ posits that preventive behaviors are most likely to be adopted when perceptions of risk and efficacy are high. Perceptions of efficacy include self-efficacy, defined as the community members’ confidence in their ability to perform the preventive behaviors in a manner satisfactory to them, and response efficacy, defined as the behaviors’ effectiveness to avert the threat. Self-efficacy is influenced by a host of factors, including the level of skill to perform the behavior,^21^ ease and convenience, access to enabling commodities (e.g., larvicide, window screens, or skin repellent in this case), household and community collaboration and other societal factors,^22^ descriptive and injunctive social norms,^23^ and periodic encouragement and feedback about the behaviors practiced,^21^ all of which increase community self-efficacy for continuing to practice these behaviors.^21^ Community self-efficacy is better described as collective efficacy, a concept that stresses the interdependent efforts and influences of community members to achieve desired results, whether working collectively or individually.^24^ SBCC may be more effective in increasing people’s efficacy if it strategically addresses these influencing factors. We explored perceived seriousness of Zika illness in the community as a proxy for risk. As a proxy for efficacy, we explored the cultural salience, perceived effectiveness, collective efficacy, and feasibility of Zika prevention recommendations in the community context.

### Selection of study sites.

In collaboration with the USAID/Guatemala and Asociación Pro-Bienestar de la Familia de Guatemala, a USAID-funded Zika-implementing partner in Guatemala, we selected two study sites: Mazatenango, Suchitepequez, a town in the Pacific lowlands 371 m above sea level, and Barberena, Santa Rosa, a town in the southeast 1,221 m above sea level. These towns are referred to in this study as the lowland town and the highland town, respectively. Zika cases had occurred in both towns according to the Ministry of Health reporting, and the partner organization was implementing Zika-related home visits and community-level intervention activities in both towns through community outreach workers or clinic staff who integrated Zika into their regular counseling on family planning, reproductive health, or antenatal care.

### Selection of study participants.

In each town, a community outreach worker who engaged urban or peri-urban communities in the organization’s intervention activities extended invitations to interested clients through convenience sampling. Invitations were made according to the following selection criteria: women aged 18 years or older who were currently pregnant, male partners aged 18 years or older of currently pregnant women, and women aged 18–30 years with a steady partner and either no children or a single child. We considered the latter group as being likely to become pregnant in the near future.

### Data collection.

Between July 27 and August 1, 2017, we conducted two focus groups with one population group per site, for a total of 12 focus groups, six per site, during mostly rainy weather. All data collection team members were experienced in qualitative methods, fluent in Spanish, and familiar with Zika. The focus group moderator was a Guatemalan national. We used a structured focus group discussion guide, touching first on the perceived seriousness of Zika, followed by three collective rank-ordering tasks of 19 cards, each depicting household or community actions. Many, although not all, of these actions represented a Zika prevention method recommended at the time. Several methods were informed by the Strategic Communication for Zika Prevention: A Framework for Local Adaptation*.*^10^ The moderator introduced the cards to the participants not as methods to prevent Zika but as a series of actions by community members and not directly by the participants themselves, to discuss during the rank-ordering tasks. Participants rank-ordered each card three times, according to its relative effectiveness for preventing Zika, according to its relative collective efficacy to be performed by community members, and according to its relative feasibility in the community context. The moderator promoted discussion on why a depicted action was ranked higher or lower than another and to shed light on disagreements among participants when applicable. All discussions were audio recorded.

Arriving participants (*n* = 68) were first asked to individually free list^25^ all the actions that people in the community currently take to avoid Zika, and their responses were recorded in writing, in the sequence in which they were given. We probed further to gain more detail on the nature of the action mentioned and to ensure that the participants mentioned all potential actions they knew of.

### Informed consent.

We obtained oral informed consent from all participants. The research protocol and the recruitment and consent scripts were approved by the Johns Hopkins Bloomberg School of Public Health Institutional Review Board in the United States (protocol 00008014) and by Zugueme, an independent ethics committee in Guatemala (protocol OFZU 1387-17).

### Data analysis.

A team of six researchers fluent in Spanish and familiar with Zika and the Guatemalan context analyzed the data, four of whom were also present for all data collection activities. We entered free-listing data into Excel and consolidated similar wordings of the same action into a single best wording. We calculated individual salience for each action mentioned by each participant and (mean) Smith’s salience^25^ for each action across all participants in each target group and at each study site. Smith’s salience is a statistical accounting of rank and frequency which describes the relative importance of items included in a mental category, in this case, actions to prevent Zika.

We transcribed focus group recordings and conducted thematic analysis on the textual data providing the context on perceived seriousness of Zika illness and on why a certain action was ranked higher or lower than another action in the card set. We developed topical codes by action, effectiveness, collective efficacy, feasibility, and relevance to Zika seriousness; we then coded the data in MaxQDA 2007 (VERBI GmbH, Berlin, Germany).

The rank order of the 19 cards was photographed and entered into Excel. The measures ranked higher have the lowest scores, and those ranked lower have the highest scores. We calculated mean ranks for each card-depicted action, by effectiveness, collective efficacy, and feasibility, in each target group and at each study site. We calculated a shared mean rank in the case of tied-ranked cards. We considered the rankings as high (ranked from 1 to 6.33), variable or moderate (ranked from 6.34 to 12.66), and low (ranked from 12.67 to 19) by dividing 19 into three approximately even intervals. To summarize the emerging theory on efficacy as shaped by the data, we rank-ordered Smith’s salience and the three sets of overall mean ranks (effectiveness, collective efficacy, and feasibility) for each of the card-depicted actions, as well as condensed the qualitative data.

## RESULTS

### Sociodemographics of study participants.

Each focus group included five to nine individuals, for a total of 70 participants, who were of Mestizo and Indigenous ethnicities. The majority of the women were homemakers (15 of 20 were pregnant women and 17 of 28 women were likely to become pregnant). The other women included three domestic workers, two food vendors, three teachers, two students, a social worker, a secretary, and a sales associate. One woman did not provide any occupation. Male partners of pregnant women included three farmers, two security guards, two mechanics, two students, a lawyer, a cook, a salesman, a supermarket employee, a credit inspector, a commercial driver, a laboratory technician, and an ornamental flower grower. One man was recently unemployed. Table 1 summarizes participants’ demographics.

**Table 1:** Demographic description of study participants*

Participant group (*N* = 68)		Range	Median	Mean
Pregnant women (*n* = 20)	Age (years)	18–36	22	23.4
	Years of schooling	0–12	9	7.2
	No. of children	0–5	1	1.1
Male partners of pregnant women (*n* = 20)	Age (years)	17–54	30	31.8
	Years of schooling	4–17	11.5	10.1
	No. of children	0–3	1	1.5
Women likely to become pregnant (*n* = 28)	Age (years)	18–30	26	25.7
	Years of schooling	2–17	11	9.9
	No. of children	0–4	1	1.2

* Two participants arrived late, and we did not have a chance to collect their demographic and free listing data; therefore, information on only 68 participants is presented.

### Perceived seriousness of Zika illness overtime.

A majority of women participants and many of the men described their community as being seriously concerned when they first heard about Zika because it appeared soon after an outbreak of chikungunya. In that outbreak, almost everyone had experienced severe symptoms of the disease, and they subsequently feared that Zika would be similar. But the milder symptoms or lack of symptoms associated with Zika reduced the public’s concern, and a parallel decline in messaging led participants to believe that the worst was over.

However, pregnant women participants expressed persisting levels of fear because of the consequences for their babies and concern that people in their communities did not believe Zika was real but rather “*inventions of the health center.*” Many male participants did not believe that they could transmit Zika in the absence of symptoms, and they thought that individuals just had to “wait it out” when symptoms were present.

Oh, it is like a dengue, a chikungunya, nothing happens, it is one illness more that does not have consequences of a different nature.—Men with a pregnant partner, lowlands site

Men cited concerns about the impact that Zika could have on pregnant women and babies, but they placed the primary responsibility for preventing Zika on women Overall, uncertainty existed about the differences between Zika, dengue, and chikungunya and whether Zika was the same as the other two diseases.

### Cultural salience of actions taken by people in the community to avoid Zika.

During the free-listing task, 68 participants mentioned 214 different actions taken by people in the community to avoid Zika. These actions were consolidated into 88 actions according to similarity. A mean of 7.06 actions were elicited per participant. Table 2 shows the first 32 elicited actions in the order of Smith’s salience.

**Table 2:** Cultural salience of free-listed community actions to avoid/prevent Zika

Actions mentioned by participants (*N* = 68)		Smith’s salience*	Participants mentioning the action, *n*
1	Use a mosquito net	0.40	45
2	Eliminate/throw away containers exposed to rain	0.31	26
3	Use skin repellent	0.24	34
4	Clean the house and areas around it	0.21	22
5	Use a condom to prevent sexual transmission	0.19	32
6	Burn or take garbage to its place	0.14	14
7	Fumigate outside the home or in the community	0.14	18
8	Burn mosquito coils at night	0.13	17
9	Wash *pilas* well	0.12	13
10	Treat *pilas* with Abate (Temephos)	0.09	13
11	Empty water from the containers you want to keep	0.09	8
12	Remove/sell bottles exposed to rain	0.08	7
13	Use clothing that covers the whole body	0.08	11
14	Eliminate tires exposed to rain	0.08	9
15	Make smoke/burn incense to shoo the mosquitoes	0.07	10
16	Abstain from sexual contact during pregnancy/when you have Zika	0.07	10
17	Fumigate the house	0.07	12
18	Wash the tires	0.06	5
19	Use plug-in tablets	0.06	6
20	Cover drums	0.06	6
21	Do not keep standing water (dirty or clean)	0.06	5
22	Keep containers clean	0.05	5
23	Pour chlorine drops in *pilas* and drums	0.05	5
24	Install screens on doors and windows	0.05	4
25	Wash drums well	0.04	5
26	Cover [small] water-holding containers	0.04	5
27	Overturn bottles exposed to rain	0.04	4
28	Cut the bush around the house	0.04	4
29	Eliminate puddles around the house	0.04	4
30	Clean/drain the water or mud around the house	0.04	4
31	Inject/vaccinate for the strong pains of chikungunya/dengue/Zika	0.04	6
32	Spray insecticide aerosols indoors	0.03	7

* Rounded to two decimal places. Salience for all elicited responses by participant group, and the field site is reported in Supplemental Table 1.

Because of the large number of actions elicited, the salience data are more meaningful in comparative terms than as individual values (Table 2). Bed net use had the highest salience possibly because of its consistent promotion for malaria control, and it was more salient for the pregnant women than the other groups, likely reflecting antenatal counseling at the time. “*Deschatarrización*,” or the elimination of containers that accumulate “*agua del cielo*” or rainwater, had the second highest salience. Participants often explained that the mosquito larvae come with the rainwater or that the rainwater attracts the mosquito, which then places its eggs and larvae in the containers. The third most salient action was skin repellent use, which was more salient for the residents of the lowland town than those of the highland town. The lowland town residents explained that the coast generally has many more mosquitoes than the rest of the country.

Among other high-salience actions was general cleaning of the house and its surroundings (fourth), which included sweeping dust away, sweeping under beds, and keeping the yard clean. Condom use to prevent the sexual transmission of Zika (fifth) was more salient for the male partners of pregnant women, followed by the pregnant women, and less salient for the women likely to become pregnant, indicating that this new Zika-specific recommendation was coming across. Although lower in salience (31st), a vaccine for Zika was mentioned in two ways: some women thought that they had already received a Zika vaccine in the arm along with other vaccines received at antenatal care, whereas others termed an injection of strong pain medicine to calm the symptoms of chikungunya a vaccine. Male participants noted that clandestine vendors sold such “vaccines” during the chikungunya outbreak.

### Perspectives on the relative effectiveness of Zika prevention recommendations.

Table 3 presents the ranking data of the card-depicted actions according to their relative effectiveness to prevent Zika, by participant group.

**Table 3:** Effectiveness ranking summary data—mean rank by participant group*

Prevention method depicted on card	Women currently pregnant	Men with a pregnant partner	Women likely to become pregnant	Overall mean rank
Removing any container-like objects from public spaces	3.42	4.75	10.25	6.14
Eliminating/emptying containers in the yard	4.25	9.13	8.56	7.31
Sweeping outdoors	5.04	4.00	9.13	6.06
Eliminating exposed tires	7.67	7.13	8.31	7.70
The steps of water container (*pila*) cleaning	7.75	3.75	6.00	5.23
Chlorinate the water in the drum (and cover)	8.25	9.75	14.00	10.67
Bed net use during pregnancy	8.88	12.13	7.38	9.46
Covering water drums	9.13	6.50	11.88	9.17
Emptying buckets	9.13	9.13	7.44	8.56
Screens for windows and doors	9.88	11.63	6.63	9.38
Temephos (larvicide) application to stored water	10.00	7.50	13.56	10.35
Skin repellent use during pregnancy	10.63	13.63	10.25	11.50
Condom use during pregnancy	11.25	15.13	6.81	11.06
Technician spraying insecticide indoors	11.38	8.63	8.69	9.56
Condom use outside of pregnancy	12.63	14.00	8.50	11.71
Family planning methods	13.75	16.50	13.13	14.46
Abstinence from sexual relations	15.50	16.13	11.06	14.23
Outdoor fogging	16.00	7.50	12.75	12.08
Wearing long sleeves	16.00	13.13	12.75	13.96

* Average of four focus groups, two per study site. Rank range 1–19. The order in which prevention methods are listed follows the rank order by women currently pregnant for which Zika-related risk would be most immediate.

Pregnant women ranked the elimination/emptying of various containers from the yard, the elimination of any container-like objects from public spaces, and sweeping outdoor areas as being high in effectiveness (3.42–5.04, Table 3) as did some of the other participants. However, the outdoor sweeping card did not show any containers being swept out—only leaves and paper being swept from the ground. Thus, the specific elimination of containers was equated with general unspecified cleanliness:

So these four [cards] that we have considered, for us are almost the same. Why? Because this is how we prevent diseases, doing cleanings and picking up the waste that is near us.—Women currently pregnant, lowlands site

This finding, along with the high salience of cleaning the house and the area around it (Table 2), reveals people’s lack of an exclusive connection between the immature mosquito stages in the water containers and Zika prevention, or a perception that mosquitoes are also harbored in dry waste.

Detailed *pila* cleaning was ranked as being high in effectiveness by all focus groups and achieved the highest rank (5.23, Table 3). With regard to the steps of *pila* cleaning, participants in all focus groups identified the use of bleach as a cleaning agent and as a means to disinfect the freshly refilled water, but not as a specific application to destroy mosquito eggs.^26^

At the end, first we brush well the pila with detergent and then I rinse it, then I pour bleach, then I rinse it again, then I clean it, I fill the pila up and pour bleach.—Women currently pregnant, highlands site

Condom use during pregnancy ranked low in nine of the 12 focus groups (moderate rank 11.06, Table 3). Many of both men and women chuckled at that card, their ranking driven by gender norms and by identifying condoms as irrelevant when one is already pregnant, viewing pregnancy as a time when women have little or no sex or as a contraindication if pregnancy were diagnosed as high risk. However, in several focus groups, and in agreement with its high salience (Table 2), individual participants testified to the effectiveness of condom use during the Zika epidemic:

She is pregnant and the only family planning method that protects her from whichever illness is the preservative… It is the only one, not any other, correct?... Because there are many family planning methods.—Men with a pregnant partner, highlands site

Family planning to delay pregnancy during the epidemic ranked generally low in effectiveness (13.13–16.5, Table 3) because most participants did not make the association with Zika prevention. However, a very small number of participants in two focus groups said that the methods were effective in helping to delay pregnancy during the Zika epidemic.

Elimination of exposed tires ranked moderately (7.13–8.31, Table 3), although participants were aware that tires led to mosquito infestation, describing the difficulty in eliminating them and coloring effectiveness by collective efficacy (Table 4). Emptying of buckets ranked variably (7.44–9.13, Table 3) because it did not solve the problem, according to participants—it only transferred standing water to puddles on the ground. This finding reveals people’s lack of awareness that *Ae. aegypti* lays its eggs exclusively on the inner walls of water containers, in contrast to other mosquito genera, which lay their eggs directly on the water (such as puddles on the ground). Water chlorination in the drum followed by covering the drum and a related card showing the hermetic covering of water storage drums ranked variably among the participants (6.50–14.00, Table 3) as being not consistently effective because mosquitoes often found their way in under the cover.

**Table 4:** Collective efficacy ranking summary data—mean rank by participant group*

Prevention method depicted on card	Women currently pregnant	Men with a pregnant partner	Women likely to become pregnant	Overall mean rank
Removing any container-like objects from public spaces	4.63	14.13	11.50	10.08
Eliminating/emptying containers in the yard	7.63	5.50	9.75	7.63
Sweeping outdoors	3.63	10.50	6.38	6.83
Eliminating exposed tires	10.63	11.38	8.00	10.00
The steps of water container (*pila*) cleaning	4.50	6.13	1.75	4.13
Chlorinate the water in the drum (and cover)	5.38	8.88	10.00	8.08
Bed net use during pregnancy	8.00	6.63	8.25	7.63
Covering water drums	6.25	6.50	6.56	6.44
Emptying buckets	8.75	7.88	4.38	7.00
Screens for windows and doors	15.13	10.88	5.06	10.35
Temephos (larvicide) application to stored water	8.00	7.88	9.63	8.50
Skin repellent use during pregnancy	8.00	13.75	7.75	9.83
Condom use during pregnancy	10.38	9.00	12.94	10.77
Technician spraying insecticide indoors	12.50	7.88	13.81	11.40
Condom use outside of pregnancy	13.00	10.38	14.69	12.69
Family planning methods	15.88	15.75	13.94	15.19
Abstinence from sexual relations	15.63	14.88	14.31	14.94
Outdoor fogging	16.63	12.50	14.44	14.52
Wearing long sleeves	15.50	9.63	12.13	12.42

* Average of four focus groups, two per study site. Rank range 1–19. The order in which prevention methods are listed is the same as in Tables 3 and 5 for easier comparison between tables.

Despite the high salience of bed nets (Table 2), their use during pregnancy ranked low in some groups and moderately overall (7.38–12.13, Table 3), while individual participants disagreed with their group depending on whether they themselves already owned and used a bed net or not. When bed nets were assigned a low effectiveness ranking, it was because of their apparent inability to stop the mosquitoes. However, no one mentioned that *Ae. aegypti* is a day-biter, which suggests that people do not differentiate between mosquito types. Skin repellent use during pregnancy received a wide range of group rankings (2.50–17.00), averaging between 10.25 and 13.63 (Table 3). Despite being recognized as effective in most discussions, participants who assigned it low effectiveness were influenced by feasibility (Table 5), citing access problems, such as cost, and the need to constantly reapply it being impractical.

**Table 5:** Feasibility ranking summary data—mean rank by participant group*

Prevention method depicted on card	Women currently pregnant	Men with a pregnant partner	Women likely to become pregnant	Overall mean rank
Removing any container-like objects from public spaces	5.88	9.38	8.38	7.88
Eliminating/emptying containers in the yard	5.88	5.75	4.63	5.42
Sweeping outdoors	6.13	9.13	4.13	6.46
Eliminating exposed tires	10.75	4.75	7.75	7.75
The steps of water container (*pila*) cleaning	5.13	4.13	3.38	4.21
Chlorinate the water in the drum (and cover)	8.63	10.63	10.13	9.79
Bed net use during pregnancy	7.25	5.75	11.00	8.00
Covering water drums	6.50	7.13	7.38	7.00
Emptying buckets	9.38	7.25	4.00	6.88
Screens for windows and doors	14.00	10.63	13.00	12.54
Temephos (larvicide) application to stored water	12.25	11.25	8.38	10.63
Skin repellent use during pregnancy	8.13	14.25	12.38	11.58
Condom use during pregnancy	8.50	11.50	12.00	10.67
Technician spraying insecticide indoors	12.00	9.63	11.38	10.98
Condom use outside of pregnancy	13.25	10.25	14.13	12.54
Family planning methods	14.88	17.13	16.63	16.21
Abstinence from sex during pregnancy	12.25	16.50	15.25	14.67
Outdoor fogging	13.63	13.88	11.00	12.83
Wearing long sleeves	15.63	11.13	11.50	12.75

* Average of four focus groups, two per study site. Rank range 1–19. The order in which prevention methods are listed is the same as in Tables 3 and 4 for easier comparison between tables.

Women in two focus groups thought that the effectiveness of measures taken in the home (bed net and condom use during pregnancy) depended on whether any measures were concurrently taken in the yard, which in turn depended on whether any measures were concurrently taken at the community level. If the health center had fogged outdoors and the community members had cleaned up the environment from containers, then the elimination or emptying of containers in one’s own yard would be more effective. And if the latter had happened, the use of bed nets and condoms in the home would in turn be more effective in reducing any residual risk. They, therefore, ordered the cards in that sequence.

If we clean indoors and not outdoors, it cancels out because the little animals are going to enter.—Women likely to become pregnant, lowlands site

### Perspectives on collective efficacy and feasibility to practice the Zika prevention recommendations.

Table 4 presents the ranking data of the card-depicted prevention measures for relative collective efficacy, whereas Table 5 presents the ranking data according to their relative feasibility.

Prevention methods that ranked quite high for both collective efficacy and feasibility were the detailed *pila* cleaning and outdoor sweeping of non-containers (Tables 4 and 5). Detailed *pila* cleaning ranked highest for collective efficacy (4.13), as it had for effectiveness (Table 3). Importantly, participants reiterated that there was a right way and a wrong way to clean the *pila* and that not everyone did it correctly; however, they expressed high collective efficacy for the correct way, according to the steps that the *pila* cleaning card illustrated, namely, with detergent, bleach, and a brush, and reiterating the final step of water disinfection with bleach on refilling if the water was not chlorinated. Cleaning the *pila* also ranked highest for feasibility (4.21), and many participants indicated that all their community members did it, from constantly and daily to three times a week to once a week—“*Who doesn’t do it?*” For some, cleaning the *pila* was considered cost effective, whereas for others, a certain cost was implied from having to buy soap and bleach, and due to an inadequate water supply*.* One group of pregnant women gave this measure a low feasibility ranking because pregnancy made it hard to bend and do the cleaning well, but the overall high rankings revealed strong underlying community norms of cleanliness and a sense of control. In reference to who actually cleans the *pila*, men generally thought the women should do it, although several men shared that they too will help with *pila* cleaning especially during a partner’s pregnancy.

The importance of maintaining a clean home was a recurring theme, and outdoor sweeping was its embodiment. Two participants indicated that *“Cleanliness is the main thing.”* However, for the general waste removal to be feasible, reliance on local municipalities was necessary. This reliance lowered the collective efficacy and feasibility of outdoor sweeping (6.83, Table 4; 6.46, Table 5).

Eliminating any container-like objects from public spaces as well as tires ranked higher in feasibility (7.88, 7.75, Table 5) and lower in collective efficacy (10.08, 10.00, Table 4) in contrast to the high effectiveness of both actions (Table 3). This was because public spaces were not under the participants’ exclusive control; broader community collaboration was needed to perform these measures in public spaces. Furthermore, participants mentioned that each town has locales known as “*Pinchazos*,” or tire repair shops that possess a large quantity of tires and take little or no action to store them properly. Households have little control over such situations and depend on the functionality of the local government’s waste services.

Emptying buckets ranked as a highly feasible action by some groups, but other participants indicated that they must store water and collect rainwater because of the lack of infrastructure supporting consistent delivery of water to their households. Emptying buckets was more palatable if it leveraged alternative uses such as outdoor washing/cleaning. It, therefore, received moderate rankings (7.00, Table 4; 6.88, Table 5).

Covering water storage drums had lower feasibility (7.00, Table 5) because of a lack of access to proper covers, lack of a proper cover, and the financial cost to cover all water storage drums. Households may opt to only cover the water that must remain clean for drinking or food preparation, as opposed to water used for less critical activities. But covering ranked higher for collective efficacy (6.44, Table 4), indicating a positive attitude that could be leveraged if the feasibility barriers could be overcome.

Bed net use during pregnancy received moderate rankings (7.63, Table 4; 8.00, Table 5), as with effectiveness (Table 3), despite its high salience (Table 2). In contrast to the women’s groups, the men in the highland town conveyed high collective efficacy (mean rank 3.5), whereas the men in the lowland town conveyed lower collective efficacy (mean rank 9.75) because of the excessive heat typical for their geographic location. The latter men pointed out how important the bed net was with so many mosquito-borne diseases to the extent that they were trying to get their children used to sleeping under a net from the start because it was much more difficult to get used to it as an adult.

Using skin repellent during pregnancy had moderate rankings (9.83, Table 4; 11.58, Table 5) because of the need for frequent reapplication and because of the cost implied. Furthermore, as a male participant explained, there was a culture clash: during pregnancy and after giving birth, new mothers are looked after by their own mothers who will not let them use repellent. Their mothers view it as a dangerously strong product that should not be used when a pregnant woman or a young mother and her new baby are “*en dieta*”† because of their vulnerable state.

Similar to the effectiveness rankings, collective efficacy and feasibility of condom use for Zika prevention during pregnancy ranked moderately (10.77, Table 4; 10.67, Table 5) primarily because of the difficulty of negotiating condom use with a steady partner. Men stated additional inhibiting factors such as cost, lack of access, embarrassment about asking for condoms at the health center despite their being free, and the persistence of *machismo:*

Participant 1: [machismo] could apply…, “oh no, I don’t use this because it doesn’t feel the same” [as without it]… Participant 3: Or “it is my woman and no one else touches her, so why use this if this is my woman.”—Men with pregnant partners, lowlands site

However, condom use during pregnancy ranked high in two focus groups and individually among various other participants, as a compromise for the sake of pregnancy.

…would be very good to utilize condoms during pregnancy…—Men with a pregnant partner, highlands site

…yes, the baby also runs the risk of Zika. We are not currently using, but, yes, I will start telling him that we should use...—Women likely to become pregnant, lowlands site

Family planning methods ranked low (14.81; Table 4; 16.2, Table 5), but were feasible for a few participants during a Zika outbreak:

…but in that sense, to be able to protect yourself and be able to have a baby… you need to think of those methods first and foremost, until the process is completed and not hearing about Zika anymore, or that the environment is calm enough to be able to carry a pregnancy well…—Women currently pregnant, lowlands site

## DISCUSSION

Our study generated insights as to which Zika prevention measures were salient to two communities and explored the perceived effectiveness, collective efficacy, and feasibility of a range of commonly promoted Zika prevention measures. Our sample sizes for collecting free-listing and focus group data were adequate to achieve salience and thematic saturation^27^ and, therefore, attain information power commensurate with our study aim.^28^

A good degree of agreement was observed between the salience of the freely elicited actions and those recommended programmatically. At least 20 of the 32 free-listed actions identified are recommended by public health authorities and partners in the LAC region.^9^ However, 20 actions are too many for the priority community audiences to consistently perform for a single public health problem, no matter how important to them, and too many for a program to promote successfully.^29^ This multitude of actions represents an inherent difficulty in the community’s response to Zika, dengue, and chikungunya, given that the actions to avoid mosquito bites and to eliminate mosquito larvae and eggs are common to all three diseases. The community needs consistent SBCC guidance and encouragement from highly trained community outreach staff to achieve high adherence for so many behaviors.

The remaining 12 salient actions for the participants are not effective for Zika prevention (e.g., mosquito coil use, cut the bush around the house, and eliminate puddles), and they represent a great deal of effort and expense on the part of the community to continue implementing them. Some of these actions may have high response efficacy against nuisance and night-time mosquitoes that are more noticeable by the community, but they are ineffective for the day-biter *Ae. aegypti*. A case in point is the high salience of using a bed net. Bed nets have high response efficacy against night-time mosquitoes, but their salience in this study highlights people’s lack of differentiation between day- and night-biters and an apparent conflation of recommendations with malaria control programs. Revised Zika recommendations do not include the use of a bed net,^9^ but the challenge remains on how to focus programmatic efforts on *Ae. aegypti* when people are bothered by many other nonvector mosquitoes at the same time.

The theory emerging from our study data is synthesized in Table 6. Although many methods had high salience, fewer methods ranked high for the other three behavioral determinants, and 10 ranked low across all 3.

**Table 6:** Emerging theory on the community’s efficacy to prevent Zika virus disease, as shaped by the data

Prevention method	Effectiveness rank*	Collective efficacy rank*	Feasibility rank*	Cultural salience rank*	Thought of as
All methods					Effective and feasible if practiced regularly and by everyone; each method complements the other
Detailed *pila* cleaning	1	1	1	9	Very salient to salient, very effective against all diseases in general, very feasible, under women householders’ full control; influenced by strong cleanliness community norms
Sweeping outdoors	2	3	3	4	
Emptying buckets	6	4	4	10.5	
Covering water drums	7	2	5	19.5	Salient, very feasible, influenced by strong water protection community norms, but not always effective—mosquitoes can find their way in
Eliminating exposed tires	5	5.5	6	13	Salient, very feasible, and effective, if in one’s own yard, and/or with good waste collection; otherwise problematic
Eliminating/emptying containers in the yard	4	10	2	2	Very salient, very effective against mosquitoes brought by rain, feasible, under the householders’ control but recurring and labor-intensive hence of lower collective efficacy
Removing any container-like objects from public spaces	3	11	7	2	Very salient, very effective against mosquitoes brought by rain, but recurring and needs community coordination, therefore, less feasible
Bed net use during pregnancy	9	5.5	8	1	Very salient, feasible for those who practice it, and effective during pregnancy depending on climate and numbers of mosquitoes, but is not a community norm for adults
Chlorinate the water in the drum (and cover)	12	7	9	23 and 19.5 (for covering)	Salient, feasible for those who practice it but covering is problematic because mosquitoes can find their way in; therefore, effectiveness is doubtful
Screens for windows and doors	8	12	14.5	23	Salient to very salient; cost, gender norms, climate, or locus of control in the health authorities inhibit feasibility and reduce practical effectiveness; willingness by many to make an exception for condom use during pregnancy
Skin repellent use during pregnancy	14	9	13	3	
Temephos (larvicide) application to stored water	11	8	10	10.5	
Technician spraying insecticide indoors	10	14	12	16	
Condom use during pregnancy	13	13	11	5	
Condom use outside of pregnancy	15	16	14.5	5	
Outdoor fogging	16	17	17	6.5	
Wearing long sleeves	17	15	16	13	
Abstinence from sex during pregnancy	18	18	18	16	
Family planning methods	19	19	19	–	

* Rank ordering of overall mean ranks/Smith’s salience.

The finding that eliminating containers exposed to rain was more salient than cleaning larger water storage containers (*pilas*) (Table 6) suggests that the latter are not seen as producing mosquitoes as much as the former. It is also compatible with the idea that mosquitoes come with rainwater. However, the opposite pattern was observed for effectiveness (Table 6). Such findings along with the finding that general unspecified house cleaning was very salient and outdoor sweeping that did not include container-like objects was ranked second highest in effectiveness (Table 6) reflect, at least in part, two notions. First, the role of mosquitoes in contagion appears to be conflated with the roles of flies and other pests, which involve breeding in dirty places and spreading filth around.^30^ Several actions brought up during free listing, such as cleaning kitchen utensils, plates, and cutlery, or keeping bathrooms clean because a mosquito “*can put its virus there*” (full data shown in Supplemental Table 1), further support this notion. Second, the frequent mention of unspecified cleaning by prevention programs perpetuates the conflation when they fail to clearly specify the type of cleaning to practice, such as the specific ovicidal cleaning of deliberate water storage containers.^9^ Unspecified cleaning is ineffective for Zika.

Furthermore, there is also conflation of recommendations from diarrheal disease control programs that promote the addition of a few drops of bleach to disinfect stored water. *Aedes aegypti* larvae are not killed by such small concentrations of bleach,^26^ yet water chlorination was consistently mentioned as the last step of an effective *pila* cleaning. Further evidence for the conflation is the manner in which two of the ranking cards were interpreted by participants. One card illustrated mixing detergent and undiluted bleach and dabbing the mixture onto the inner walls of a *pila*, waiting 10 minutes, scrubbing the container with a brush, and rinsing it.^26^ The other card illustrated dabbing bleach onto the container wall of a drum, just above the water line, waiting 15 minutes, and then covering the container.^31^ Several programs and organizations in the LAC region have promoted both methods at various times as being effective for directly destroying *Ae. aegypti* eggs.^32,33^ Neither picture was recognized by the participants as an ovicidal technique, indicating that people were neither aware of this method nor were they knowledgeable about where *Ae. aegypti* eggs are found. Participants interpreted the first picture as the correct steps to clean a *pila* well, and the second picture as the steps to disinfect water by adding a few drops of bleach directly into it after the cleaned container has been refilled, followed by covering. Because both of these actions had also been free-listed by the participants, we retained them in the card set for the ranking activities and used them according to the participants’ interpretation. Hence, in the Results section, the picture depicting the application of detergent and bleach in a *pila* is referred to as “The steps of water container (*pila*) cleaning” and the picture depicting the application of bleach to a drum is referred to as “Chlorinate the water in the drum.” With regard to the low-ranking methods (Table 6), cost, climate, gender norms, or locus of control outside the individual were cited as reasons for low ranking.

In the end it is easier to abstain than to obligate the State to fog you.—Men with a pregnant partner, lowlands site

Sexual abstinence for Zika prevention ranked low; however, several women voiced higher collective efficacy to negotiate abstinence during pregnancy than condom use:

Participant 2: It is easier that they accept our saying no. Participant 6: …Because it feels that [having sex] is not the most important… that our baby needs protection… This is why I think that it may not be difficult to avoid [having sex].]— Women likely to become pregnant, lowlands site

Sexual abstinence in Guatemala is sometimes part of the *dieta* and was a form of support given by husbands to their wives in another study.^34^ Therefore, it might be worth exploring by programs whether abstinence is self-efficacious for some audiences, although sexual transmission of Zika may be a small portion of overall transmission in autochthonous mosquito-borne transmission settings by some estimates,^35^ whereas higher by others.^36^ The absolute risk of sexual transmission of Zika has not been quantified, but it appears to be small.^37^

Gender and relationship norms were the rationale for the low rankings of family planning methods during a Zika outbreak. Women participants indicated that many men did not permit their partners to use such methods, and some women who used them did so secretly. This situation highlights a lack of discussion and equity in decision-making among couples with respect to family planning in general. Gender and relationship norms influenced the low rankings of using condoms before or during pregnancy; however, participants of both genders expressed a clear desire to protect their unborn or future children. This desire may be leveraged by programs to increase condom use for Zika prevention during pregnancy or to delay pregnancy until after an outbreak.

The results of the present study point to several recommendations for Zika prevention behavior change programs. Although the number of preventive behaviors to promote needs to be reduced, this reduction is challenging when behaviors pertain to a vector that breeds in a broad variety of habitats and is a day-biter. Reduction could be attained by bringing greater precision to the recommended behaviors. The examples listed below are based on our findings. Additional qualitative research can further help programs fine-tune and focus the behaviors they promote, based on the clarification and specificity that communities indicate as being most required.

1. Abandon the term “to eliminate standing water” from program recommendations. Standing water also means puddles, open sewage, flooded fields, and other habitats not attractive to *Ae. aegypti.*^38^ Instead, specify that the type of standing water to be eliminated or protected is within containers.
2. Abandon the term “cleaning” from program recommendations. Specify the targeted steps and actions instead. Ordinary scrubbing with a brush during the typical cleaning process at best dislodges the eggs but does not kill them.^26^ Clarify and demonstrate the use of detergent and household bleach, or bleach alone, as an ovicide against *Ae. aegypti* eggs.^26,31,32^ Demonstrate where the eggs can be found and what they look like. Demonstrate the difference between dabbing and wiping bleach and point out the need to wait a specified number of minutes for the bleach to have effect.
3. Distinguish between mosquito types^38^ and identify the types of mosquitoes for which high salience actions, such as bed nets, burning coils, incense, smoke, or puddle elimination, are effective.
4. Clarify that rainwater and tap water can become infested with larvae because *Ae. aegypti* lays eggs equally well in a container in which rainwater has accumulated and in one where tap water is stored (“*agua del cielo*” versus “*agua de la llave*”).
5. In this study context, the term “repellent” was allocated two meanings by study participants: skin repellent and spatial repellent. Recommendations should, therefore, be clear on which type of repellent to use. Similarly, “vaccines” have two meanings, referring to vaccines, but also to injections of any type. Health communicators should, therefore, be alerted on how to use the term “vaccine” and trained to recognize its meaning when used by their audiences, so as to minimize the community’s impressions that a vaccine for Zika exists.
6. Where possible, increase the effectiveness and feasibility of recommended methods, by improving the enabling technology, such as designing a mosquito-proof water storage container, a mosquito-proof lid, and window screens compatible with the local house construction, and adapting mosquito nets and insecticide-treated materials for day-time use, especially for pregnant women.
7. Increase self-efficacy for condom use among young adults who are not planning a family.
8. Increase self-efficacy for condom use at the suspicion of pregnancy and during pregnancy, capitalizing on the concern of the fathers-to-be for their unborn baby. Encourage couple communication on this topic at the first suspicion of pregnancy, and ideally earlier. To influence pregnant women, also targeting their mothers and mothers-in-law is essential because they play an important role caring for their pregnant daughters and generally make decisions about how to best protect them from mosquitoes or other adversities during that period.
9. Lastly, engage community leaders, government organizations, the private sector, and community-based civil society organizations to take an active role in *Ae. aegypti* control and to be vocal about their collective efforts to reduce larval habitats in communal areas; intersectoral collaboration has an important role to play in vector-borne disease prevention.

## Supplemental table

Supplemental materials

## Footnotes

† A set of dietary and other restrictions including “*tapado*” or the covering of the body with warm clothing and sexual abstinence during pregnancy and after giving birth, prevalent in the Guatemalan traditional culture.
