# Supplementary material for: Community Perspectives on Zika Virus Disease Prevention in Guatemala: A Qualitative Study
Source: Am J Trop Med Hyg. 2020 Feb 24;102(5):971–81. doi: 10.4269/ajtmh.19-0578 (PMC7204582; doi:10.4269/ajtmh.19-0578)
Supplement: Supplementary file 1 [file tpmd190578.SD1.pdf]

ANNEX: Supplementary Tables

Annex 1: Cultural Salience of 88 elicited Zika prevention actions, by participant group and field site

Actions mentioned by 68 participants		Overall Mean Salience	Lowlands			Highlands			Number of participants mentioning the action
			Men with a pregnant partner	Women currently pregnant	Women likely to become pregnant	Men with a pregnant partner	Women currently pregnant	Women likely to become pregnant	
1	Use a mosquito net	0.40	0.40	0.60	0.55	0.38	0.42	0.12	45
2	Eliminate/throw away containers exposed to the rain	0.31	0.47	0.18	0.34	0.36	0.31	0.25	26
3	Use skin repellent	0.24	0.49	0.08	0.40	0.21	0.13	0.19	34
4	Clean the house and all around it	0.21	0.18	0.28	0.17	0.23	0.10	0.26	22
5	Use a condom to prevent sexual transmission	0.19	0.40	0.23	0.13	0.08	0.38	0.08	32
6	Burn or take garbage to its place	0.14	0.11	0.05	0.12	0.20	0.04	0.25	14
7	Fumigate outside the homes or in the community	0.14	0.32	0.03	0.03	0.11	0.04	0.28	18
8	Burn mosquito coils at night	0.13	0.10	0.09	0.01	0.23	0.19	0.16	17
9	Wash pilas well	0.12	0.10	0.17	0.18	0.07	0.00	0.18	13
10	Treat pilas with Abate	0.09	0.15	0.05	0.01	0.04	0.03	0.22	13
11	Empty the water from the containers you want to keep	0.09	0.00	0.23	0.05	0.03	0.09	0.11	8
12	Remove/sell bottles exposed to the rain	0.08	0.21	0.00	0.06	0.00	0.08	0.17	7
13	Use clothing that covers the whole body	0.08	0.08	0.06	0.09	0.19	0.11	0.00	11
14	Eliminate tires exposed to the rain	0.08	0.00	0.07	0.07	0.15	0.00	0.15	9
15	Make smoke / burn incense to shoo off the mosquitoes	0.07	0.05	0.05	0.09	0.21	0.00	0.03	10
16	Abstain from sexual contact during pregnancy/when you have Zika	0.07	0.16	0.09	0.10	0.10	0.00	0.00	10
17	Fumigate the house	0.07	0.03	0.02	0.23	0.03	0.09	0.00	12

18	Wash the tires	0.06	0.00	0.00	0.06	0.11	0.00	0.13	5
19	Use plug-in tablets	0.06	0.00	0.00	0.00	0.15	0.06	0.12	6
20	Cover drums	0.06	0.00	0.00	0.00	0.09	0.08	0.14	6
21	Do not retain standing water (dirty or clean)	0.06	0.00	0.00	0.00	0.08	0.00	0.18	5
22	Keep containers clean	0.05	0.00	0.00	0.07	0.06	0.10	0.08	5
23	Pour chlorine drops in pilas and drums	0.05	0.11	0.00	0.08	0.00	0.00	0.08	5
24	Install screens on doors and windows	0.05	0.09	0.00	0.11	0.08	0.00	0.00	4
25	Wash drums well	0.04	0.00	0.03	0.00	0.06	0.05	0.10	5
26	Cover [small] water holding containers	0.04	0.00	0.00	0.00	0.00	0.12	0.11	5
27	Overturn bottles exposed to the rain	0.04	0.00	0.14	0.00	0.06	0.00	0.05	4
28	Cut the brush around the house	0.04	0.00	0.00	0.05	0.05	0.00	0.09	4
29	Eliminate puddles around the house	0.04	0.11	0.07	0.08	0.00	0.00	0.00	4
30	Clean/drain the humidity or mud around the house	0.04	0.00	0.00	0.04	0.06	0.00	0.09	4
31	Inject/vaccinate for the strong pains of chikungunya/ dengue/Zika	0.04	0.01	0.07	0.08	0.02	0.03	0.01	6
32	Spray insecticide aerosols indoors	0.03	0.00	0.07	0.02	0.06	0.02	0.03	7
33	Store your containers safely	0.03	0.08	0.00	0.05	0.00	0.00	0.06	3
34	Keep good general hygiene to avoid diseases	0.03	0.13	0.00	0.00	0.02	0.10	0.00	3
35	Keep good food hygiene	0.03	0.06	0.08	0.00	0.00	0.00	0.06	3
36	Empty / do not keep water in drums and tanks	0.03	0.00	0.10	0.08	0.00	0.00	0.00	2
37	Place containers upside down	0.03	0.00	0.00	0.06	0.10	0.00	0.00	3
38	Take acetaminophen (for the fever, pains)	0.03	0.00	0.10	0.01	0.06	0.00	0.00	4
39	Fill in tires with soil	0.02	0.00	0.00	0.00	0.06	0.00	0.07	2
40	Eliminate plastic bags	0.02	0.00	0.00	0.03	0.06	0.06	0.00	3

41	Remove/bury food left overs	0.02	0.00	0.00	0.00	0.03	0.00	0.09	3
42	Vaccinate yourself against Zika, tetanus, flu and other diseases during pregnancy	0.02	0.00	0.00	0.00	0.06	0.08	0.00	2
43	Do not have flower pots out in the open	0.02	0.11	0.00	0.05	0.00	0.00	0.00	2
44	Keep clothing and towels clean	0.02	0.00	0.00	0.00	0.00	0.00	0.10	2
45	Eradicate the mosquito	0.02	0.18	0.00	0.00	0.00	0.00	0.00	2
46	Chlorinate, boil or purify drinking water	0.02	0.00	0.08	0.01	0.00	0.00	0.03	4
47	Cover buckets holding water	0.02	0.13	0.00	0.00	0.04	0.00	0.00	2
48	Eliminate buckets	0.02	0.00	0.00	0.00	0.08	0.00	0.03	2
49	Wash well/keep good hygiene of dishes	0.02	0.11	0.00	0.00	0.04	0.00	0.00	2
50	Keep baby and children areas clean	0.02	0.00	0.00	0.00	0.00	0.00	0.09	2
51	Cover drains	0.02	0.00	0.00	0.08	0.00	0.00	0.01	2
52	Go to the health center if you have symptoms	0.02	0.00	0.01	0.00	0.08	0.01	0.00	4
53	Use agrochemical/ veterinary insecticides	0.02	0.00	0.00	0.00	0.04	0.00	0.05	3
54	Keep trash cans covered/closed	0.02	0.00	0.09	0.00	0.00	0.00	0.02	2
55	Keep children away from puddles, sewage, mud	0.02	0.00	0.00	0.00	0.00	0.06	0.03	2
56	Keep out of humid places / grass	0.01	0.00	0.00	0.08	0.00	0.00	0.00	1
57	Clean out drains	0.01	0.00	0.00	0.00	0.00	0.09	0.01	2
58	Prevent pregnancy during the epidemic	0.01	0.00	0.02	0.05	0.01	0.00	0.00	4
59	Fill in flowerpots with soil	0.01	0.00	0.00	0.00	0.00	0.00	0.06	1
60	Wash plastic bags	0.01	0.00	0.00	0.00	0.07	0.00	0.00	1
61	Clean the bathrooms	0.01	0.00	0.03	0.00	0.00	0.00	0.03	2
62	Keep the streets clean	0.01	0.00	0.08	0.00	0.00	0.00	0.00	1
63	Use oils/pomades on the skin to prevent mosquito bites	0.01	0.00	0.00	0.00	0.03	0.00	0.03	2
64	Eliminate out of use tanks/drums/barrels	0.01	0.09	0.00	0.00	0.00	0.00	0.00	1
65	Sleep with clothing or sheets	0.01	0.05	0.00	0.00	0.03	0.00	0.00	2

	covering the whole body								
66	Install sewage pipes to avoid water pouring in the open	0.01	0.00	0.00	0.00	0.00	0.07	0.00	1
67	Pour motor oil in sewage wells	0.01	0.00	0.00	0.00	0.00	0.07	0.00	1
68	Keep your water containers clean	0.01	0.00	0.01	0.00	0.00	0.06	0.00	2
69	Keep good personal hygiene	0.01	0.00	0.00	0.00	0.00	0.00	0.04	2
70	Close the windows when it's becoming night	0.01	0.06	0.00	0.00	0.01	0.00	0.00	2
71	Spray gardens/animal pens with aerosol insecticides	0.01	0.00	0.05	0.01	0.00	0.00	0.00	2
72	Cover pilas	0.01	0.00	0.00	0.00	0.05	0.00	0.00	1
73	Burn discardable containers	0.01	0.00	0.00	0.05	0.00	0.00	0.00	1
74	Channel sewage to a mosquito protected place	0.01	0.00	0.00	0.00	0.05	0.00	0.00	1
75	Use cream on the mosquito bite to eliminate the virus	0.01	0.00	0.06	0.00	0.00	0.00	0.00	1
76	Teach our children good personal hygiene	0.01	0.07	0.00	0.00	0.00	0.00	0.00	1
77	Educate and share information	0.01	0.00	0.00	0.02	0.00	0.00	0.02	2
78	Sleep with a fan	0.01	0.06	0.00	0.00	0.00	0.00	0.00	1
79	Kill the mosquitoes (with your hand)	0.01	0.00	0.00	0.00	0.00	0.05	0.00	1
80	Use a plug-in racquet	0.01	0.00	0.00	0.00	0.00	0.00	0.03	1
81	Wash bottles	0.01	0.00	0.00	0.00	0.03	0.00	0.00	1
82	Disinfect mosquito bite with alcohol	0.01	0.00	0.00	0.03	0.00	0.00	0.00	1
83	Stop midday naps and keep yourself moving to prevent the mosquitoes from biting you	0.01	0.00	0.00	0.03	0.00	0.00	0.00	1
84	Bathe 3x/day to keep healthy (and from the heat)	0.00	0.00	0.03	0.00	0.00	0.00	0.00	1
85	Do not come close to people with Zika	0.00	0.00	0.00	0.00	0.03	0.00	0.00	1
86	Use the female condom	0.00	0.04	0.00	0.00	0.00	0.00	0.00	1
87	Go to the doctor more frequently	0.00	0.00	0.00	0.00	0.01	0.00	0.00	1
88	Lower a curtain over the house entrance to block mosquito entry	0.00	0.00	0.00	0.00	0.01	0.00	0.00	1
